# The Roles of Psychological Capital and Gender in University Students’ Entrepreneurial Intentions

**DOI:** 10.3389/fpsyg.2020.615910

**Published:** 2021-01-15

**Authors:** Clara Margaça, Brizeida Hernández-Sánchez, José Carlos Sánchez-García, Giuseppina Maria Cardella

**Affiliations:** University of Salamanca, Salamanca, Spain

**Keywords:** entrepreneurship, gender, psychology, intention, Portugal

## Abstract

Universities increasingly play an important role in entrepreneurship, which has contributed to gender equality in the business world. The aim of this study is to establish a causal model of entrepreneurial intentions and explore it by gender, based on the dimensions of the Theory of Planned Behavior, and how these are mediated by the individuals’ resilience and psychological well-being. The previous work experience was considered as one of the control variables, in order to analyze whether this influence the entrepreneurial intention. With a convenience sample of 644 Portuguese students, Structural Equation Modeling (SEM) was used. For a better understanding, multivariate analyses were performed and each one was individually reported, and for appropriate comparisons by gender, the *t*-student test was used. The comparison of means, between genders, showed that there are differences only between perceived behavioral control, subjective norm, and entrepreneurial intention, with women scoring the highest values, and psychological well-being, with men scoring the higher. A serial mediation path was performed, and psychological resilience was found to mediate a significant effect between perceived behavioral control and intention in females, but not in males. It also mediates a significant effect between attitude and intention in females, but not in males. These results show that attitude is a determining factor for females to become entrepreneurs. Finally, after discussing the results, theoretical and practical contributions are analyzed, with regard to the field of entrepreneurship in Portugal, and alternatives are pointed out for a more entrepreneurial future, reinforcing the role of higher education institutions.

## Introduction

The 2008 economic and financial crisis led to a sharp decrease in investment levels, with significant consequences for companies and people, all over the world. In 2013, Portugal started an expansion cycle, in which 2015 stands out as the best year for entrepreneurship in Portugal (GEM, 2019). Entrepreneurship is a growing phenomenon worldwide, not only because entrepreneurial activity contributes to job creation but also because it contributes to the sustainability of the competitiveness of a country’s economic activity.

As a crucial key for the transmission and dissemination of knowledge, Universities are considered a mechanism to improve economic growth (Audretsch and Caiazza, 2016), helping the development of potential entrepreneurs. This can help a country, here in particular Portugal, to reduce unemployment levels and increase entrepreneurial activity. Universities are generally seen as a driving factor, especially for young people entering the labor market; however, these are also seen as a complex issue. On the one hand, due to the vast number of areas of education and occupational approaches and, on the other, to the vast differences between individuals who are part of this branch of education.

In this study, we will focus our approach on the second topic mentioned, specifically gender differences. Entrepreneurship is portrayed in the literature as a male-dominated field (Markussen and Røed, 2017), which means that gender is a highly complex variable and moderates the individual’s behavior and intentions in becoming an entrepreneur (Markussen and Røed, 2017; Guzman and Kacperczyk, 2019). Although Universities can be a starting point for an individual’s decision to become an entrepreneur, we argue that the variability of entrepreneurial potential at the heart of the academy, by gender, must also be understood (Bergmann et al., 2018; Dilli and Westerhuis, 2018).

In Portugal, entrepreneurship is seen as the third mission of universities, as it aims to reflect their contributions to society. Currently, data from reports on entrepreneurship in Portugal assess the entrepreneurial intent of the university population (e.g., Global University Entrepreneurial Spirits ’Student Survey, 2018); however, do not study gender differences in depth. Three important Portuguese universities (University of Aveiro, University of Porto and University of Minho) created the Entrepreneurship Observatory, whose objective is to characterize entrepreneurship ecosystems, in order to understand their evolution; this mechanism seeks to make an analysis upstream and not downstream, not fully covering the characteristics of the entrepreneur or potential entrepreneur from the theoretical point of view (Santos et al., 2013).

This study seeks to answer to this gap, and the aim is to analyze the entrepreneurial intention in a Portuguese university context, and, more specifically, to understand how gender differences affect this factor. In this way, a mapping of the causal relation of psychological variables with the entrepreneurial intention will be made, in both male and female; later, it will be discussed how this relates to the situation of entrepreneurship development in Portugal.

Entrepreneurial activity is understood as being a process that develops over time, and it has a beginning long before the moment when the individuals create their company. Thus, like any other human behavior, it requires planning and understanding of the process of intention and decision making to become a business person (Loiola et al., 2016). Accordingly, the entrepreneurial intention is seen as a previous and determining element for the realization of a new enterprise, it is assumed to be the first step in the formation of new businesses behavior (Krueger, 2009; Margaça et al., 2020).

Although commonly studied in several areas (e.g., health), intrinsic psychological variables, such as resilience, are rarely included in models of intention. This is a huge gap in the literature, considering that they are variables that influence the decision to create new businesses (Lindsay, 2014). In addition, they are also cited as a reason for the creation of companies in many countries, such as Germany, and especially in females (GEM, 2019).

Thus, this study aims to 1—establish a causal model for entrepreneurial intentions applicable to the Portuguese and university context and analyze it by gender and 2—explore what the role some psychological variables play in the intention of being an entrepreneur in university students and by including them in a new causal model to understand how future entrepreneurship-oriented initiatives are explained by the current situation in the country (OECD, 2019). Finally, the study will analyze the models separately by gender, which will allow to better visualize difference and similarities, meaning it goes explaining genders as models.

## Theoretical Background and Conceptual Model

### The Role of Gender in Entrepreneurship

The definition of an entrepreneur is a strong challenge, as it is necessary to attend to the idiosyncrasies and heterogeneity of each one (Margaça et al., 2020). Thus, the concept of entrepreneurship is also not based on a static profile of people and interests (Mwatsika et al., 2018) and described as *a maturing strand of enquiry* (Jennings and Brush, 2013). It is seen as a set of individual (Kerr et al., 2018) and group (Parker, 2018) actions, which lead to the creation of new ventures that may vary according to the assessment of interests and opportunities. The literature points to a set of differences when an entrepreneurial initiative is created by groups, namely, with regard to gender (Nwankwo et al., 2012; Lima et al., 2016; Pérez-Quintana et al., 2017). It is mainly Psychology that describes strong empirical evidence that the structure of male entrepreneurship is different from the structure of female entrepreneurship, both in building business (Kelley et al., 2017) and in the goals they hope to achieve (Minniti, 2009; Marlow and Dy, 2017). These data allow us to affirm that there is a male and a female pattern in the way of making entrepreneurship happen. Some studies (Marques and Moreira, 2013; Kelley et al., 2017) show that men are more interested in Engineering and Technology, while women opt for the social aspect (Barbosa et al., 2011). The differences between both in terms of objectives, business perceptions, and resilience are also evident (González-López et al., 2019). On the one hand, these differences can be seen as positive, in the sense of a broader contribution and in different business sectors, as well as for the development of society (Brush, 2006). On the other hand, these differences are seen as gender stereotypes, that is, the business world is seen as belonging to the male universe, which increases the favorability of male models of behavior (Lewis, 2006; Feder and Niţu-Antonie, 2017). Based on this, we are allowed to argue that these evidences influence how the act of entrepreneurship is perceived by men and women.

The basis of the intention to undertake is seen as a different experience between men and women, as the perception and cognition of both (Hyams-Ssekasi et al., 2019) lead to a different path in their development. This fact has attracted interest from researchers in this field on why people become entrepreneurs and what is the role of gender in increasing entrepreneurship.

Literature assumes that intention is influenced, for instance, by personal variables and is the one that better predicts and anticipates a given behavior. Entrepreneurial intention (EI) is presupposed as being the first step in new business formation and is a deliberately designed behavior (Krueger, 2009). EI is a conscious state of mind that directs attention toward a specific goal or pathway in order to achieve the stated ambitions (Liñán and Fayolle, 2015). Recently, it has been pointed out as being a cognitive representation of the actions to be implemented by individuals who want to establish new companies (Paço et al., 2015). According to Loiola et al. (2016), the orientation of an action to a new venture can be influenced, on the one hand, by interpersonal relationships, which can provide economic, social and informational resources. Secondly, it can be influenced by cultural aspects, such as the group’s acceptance and approval of the group to certain economic activities, values, and principles (Balog et al., 2014). In general, it can be said that personality and psychological competences, as well as the environment, affect individuals’ intention to become an entrepreneur. It is this set of variables that will be considered to analyze gender differences.

## Male vs. Female: Who is More Entrepreneurial?

### The Role of Personal Perceptions

According to a literature review, it is evident that there is an inequality in relation to gender and entrepreneurship. The characteristics associated with male are seen as better adjusted to the creation of the business itself, especially with regard to motivation, attitude, and behavior (Caliendo et al., 2015). Minniti (2009) states that the difference between men and women is in their personal and entrepreneurial character, as they create businesses in different areas, stipulate different goals and the way they organize the businesses is very specific. It reveals that the differences are based on the particularities of the personality and on their psychological skills. Differences in personality, principles, and moral values lead men and women to choose different professional activities and to prefer a different organizational format. Studies also suggest that men and women differ in terms of entrepreneurial orientation and that these differences are able to explain their preferences and behaviors (Ngek and van Zyel, 2016). The 2016 World Economic Forum report describes Portugal as a country where inequality between men and women decreases from year to year, occupying Portugal 31st place in the ranking of gender differences between 142 countries. In terms of the percentage of men–women, Portugal had an evolution in parity compared to previous years, with the overall active population in Portugal having 9.2% men and 6.1% women entrepreneurs (GEM, 2019). According to the Mastercard Index of Women Entrepreneurs (2019), Portuguese women are in the top 10 worldwide. Some recent studies (e.g., Oliveira et al., 2013; Fernandes et al., 2018) corroborate that the Portuguese male students have a higher percentage toward the intention or determination to be entrepreneurs. This result is also common in other countries (e.g., Gupta et al., 2008; Ventura and Quero, 2013; Ward et al., 2019); therefore, we consider to expect the same output in this study.

Personal perceptions of controllability and self-efficacy related to a certain behavior strongly influence the perceptions of situational risks, as well as the intentionality and decision making in becoming an entrepreneur (Yates and Stone, 1992; Krueger and Brazeal, 2017). Considering evidence, it is expected in this study to find differences in the average of perceptions between male and female; however, the same is not expected in relation to the causal predictions to intention between both. In order to explore the perception of entrepreneurial behavior in students, we will use the Perceived Behavioral Control (PBC). This will make it possible to study both the controllability and the effectiveness of the respondents. Supported by Ajzen (1991), PBC is a determinant of both behavioral intention and of the behavior itself. On a conceptual basis, PBC is similar to self-efficacy—both constructs refer to the person’s belief that the behavior in question is under his or her control. However, operationally, PBC is often assessed by the ease or difficulty of the behavior, while self-efficacy is operationalized by the individual’s confidence in being able to carry out the behavior in the face of extenuating circumstances (Ajzen, 2002). Controllability refers to a person’s assessment of the ease or difficulty of becoming an entrepreneur, which means a person’s belief or perception about executing and controlling a determined behavior. In addition, it is important to emphasize that these factors can be affected by “exogenous influences” (Souitaris et al., 2007).

H1: For males and females—PBC has a significant and positive effect on EI, which is not significantly different from each other.

The attitude toward a behavior refers to the degree of evaluation—favorable or unfavorable—in relation to this behavior (Ajzen, 1991). In the field of entrepreneurship, the attitude toward creating own business is usually defined as “the difference between perceptions of personal desirability in becoming self-employed and employed” (Souitaris et al., 2007, p. 570). Several authors (e.g., Ajzen, 1991; Goethner et al., 2012; Fernandes and Proença, 2013; and many others) point out two components of the attitude: (1) affective/experiential—feelings or emotions (e.g., joy, satisfaction) and drives engendered by the perspective of performing a behavior and (2) instrumental/cognitive—beliefs, thoughts or rational arguments. This suggests that entrepreneurial behavior is a very complex interaction between predispositions (Zhao et al., 2010; Zhang et al., 2019) perceptions (Arenius and Minniti, 2005), and competences. Taking these two components into account, according to Tavares et al. (2019), it is possible to affirm that higher levels of psychological well-being positively influence the meaning at work. The individual’s attitude toward entrepreneurship (ATE) is thus determined by salient beliefs in relation to behavior—behavioral beliefs—and by personal assessment of the consequences of this behavior. Several authors (e.g., Liñán and Chen, 2009; Lopes, 2010) assume that experience, education, and human capital, that is, individual skills, cleverness, and competences, and other demographic variables influence the formation of entrepreneurial intention. Studies that related Theory of Planned Behavior and entrepreneurship concluded that both ATE and PBC are significant predictors of intention. Others have tried to separate the components of ATE and PBC to examine their relative importance in predicting intention. There is evidence that gender has a significant, albeit weak, effect on ATE and PBC (Haus et al., 2013). In this study, we do not expect to achieve significant differences between men and women. As university students, exposure to knowledge and academia is expected to improve your skills and insights on what it takes to become an entrepreneur.

H2: For males and females—ATE has a significant and positive effect on EI, which is not significantly different from each other.

### The Role of Psychological Capital

Psychological capital refers to a person’s mental state, who exhibits positive organizational behaviors and demonstrates high job performance (Costa and Neves, 2017). Psychological capital is related to the achievements and well-being of individuals. This evidence, when developed, will determine the existence (related to the entrepreneurial intention), the development and prosperity of a business (Costa and Neves, 2017; Darvishmotevali and Ali, 2020). In recent years, academy has begun to study well-being as an important entrepreneurial outcome, focusing on the psychological and resilient coping mechanisms that can affect entrepreneurs (Uy et al., 2013, 2017; Stephan, 2018). Several empirical studies (e.g., Diener et al., 2009; Peters et al., 2018; Stephan, 2018) demonstrate that the entrepreneurs’ well-being influences the cognitive processes involved in a conscious behavioral choice, such as goal setting. Thus, it is also possible to affirm that well-being influences the decision to become entrepreneur, and the direction, intensity, and persistence in the establishment and pursuit of entrepreneurial goals. A study of women entrepreneurs in the United States (O’Hare and Beutell, 2020), for instance, revealed how they emphasized the need for autonomy and flexibility (Sánchez-Cañizares and Fuentes-García, 2010), challenge, feelings of accomplishment, and well-being. The narratives of these women entrepreneurs help us understand the factors that motivated to start their business and the importance of the business for their overall well-being (O’Hare and Beutell, 2020). Other studies show the variability of the reasons for starting an enterprise, between genders, stating that men are mainly looking for profits (Maes et al., 2014). The literature makes it possible to state that motivational factors influence decision making to become an entrepreneur (Williams and Williams, 2011). GEM (2019) mentioned that 48% of Portuguese women entrepreneurs are driven by pull factors, against 27% driven by necessity. In other words, Portuguese women have an intrinsic motivation to initiate the entrepreneurial activity, explore the opportunity on their own incentive, are more motivated, and do what they want in order to also guarantee well-being. Other studies also suggest that men and women differ in terms of entrepreneurial orientation and these differences are able to explain their preferences and behaviors (Neneh et al., 2016). The Psychological Wellbeing (PWB) of workers in general can be perceived as being a multidimensional psychological construct, assimilated by fulfillment and commitment to work and affective commitment to the organization (Siqueira and Gomide, 2004; Siqueira and Padovam, 2008). Self-employed workers have higher levels of vitality and feel positively energized and cognitively more active, which translates into a better perception of health (GEM, 2019). In this way, we expect to find differences between both genders in relation to the PWB, with regard to controllability and attitude.

H3: PWB mediate the positive effect of PBC on intention, which is stronger in females.

H4: For males and females—PWB mediate the positive effect of ATE on EI, which is not significantly different from each other.

### The Role of Individual Skills

Some studies (e.g., Borges et al., 2016) report that entrepreneurs make subjective perceptions of the social environment. According to these authors, it is the social context that influences the entrepreneur in the development of strategies, and therefore, it has an important role in determining the results. This element can be perceived in different ways, when referring to gender. Portugal does not escape this trend, because there are clear phenomena of horizontal and vertical segregation, different degrees of vulnerability to unemployment, and precarious and atypical forms of employment and variable propensities to create own jobs (Casaca, 2012; Marques and Moreira, 2013), which tend to operate to the disadvantage of women, in particular. Policies aimed at promoting the entrepreneurship of women are still relatively incipient in most European Union member states (Instituto para o Fomento e Desenvolvimento do Empreendedorismo em Portugal [IFDEP], 2014, p. 33). The unequal involvement of women and men in entrepreneurial activities depends on two main factors: (1) contextual obstacles—educational choices in the formal education system and dominant representations of femininity, science and innovation and (2) economic obstacles—requiring the innovation sector to make a substantial investment and women appear to be less credible than men in terms of financing (European Commission, 2014). Since it is difficult to measure perceptions, we chose to use the subjective norm (SN), in order to infer how the social pressure that individuals can feel can influence the decision to become an entrepreneur. Some studies (Hartman and Hartman, 2008; Leroy et al., 2009) suggest that, in certain contexts, women may be more strongly motivated by social pressures than men. Hartman and Hartman (2008) found that normative beliefs are more important to influence women’s occupational intentions in an activity dominated by men. Women tend to value more the opinion of the social environment where they are inserted when deciding whether or not to become an entrepreneur. The Ease of Doing Business Index (2020) ranked Portugal in 63 out of 190 economies and presents the same results for men and women results in terms of ease of business creation. Previous research (e.g., Verheul et al., 2006) offered little empirical evidence that there is more social pressure for men to become entrepreneurs than women. Indeed, literature presents no significant gender differences in the normative opinion of others to become an entrepreneur. To summarize, both are equally stimulated by the social milieu to make the decision to become entrepreneurs. However, based on the study of Leroy et al. (2009), we believe that SN have a more important role in stimulating women entrepreneurship, who also want guarantee their well-being.

H5: For males and females—SN toward entrepreneurship has a significant positive effect on EI, which is not significantly different from each other.

H6: For males and females—PWB mediates the positive effect of SN on EI, and the effect is significantly stronger in females.

The transformations in the global economy and in the world of work have led to new forms of labor relations and thus demand new personal and professional characteristics. In a highly competitive market, it is not enough to have technical skills and expertise in line with what is required. Increasingly, behavioral competencies have represented a major differential for the success and failure of a career and a business of its own (Shin and Kelly, 2015). Faced with difficulties and, through Psychological Resilience (PsyResil), people are able to renew and dedicate themselves to achieve success, dealing with previous mistakes as a learning and seeking the necessary knowledge for good management and market vision (Kolar et al., 2017). PsyResil is considered as an interactive process between the person and the social environment (Rutter, 1987; Rutter, 2012); it is an important and essential feature in the decision to become an entrepreneur, as well as guiding success in maintaining their venture (Cheng et al., 2020).

In the current competitive business world, PsyResil is a predictor of business success at all stages of the entrepreneurial activity, and an important personal quality for entrepreneurs, both men and women (Bullough and Renko, 2013). The literature points out that female entrepreneurs exhibit different characteristics of entrepreneurial resilience when compared to men (Markman et al., 2005). Women from decision making tend to be more psychologically resistant and ready to face challenges in a stable way. In addition, it is believed that women entrepreneurs who demonstrate entrepreneurial resilience are willing to work harder to achieve all their goals, to adapt quickly to changes, in order to create and take advantage of new business opportunities (Loh and Dahesihsari, 2013).

According to National Statistics Institute (INE) data, in October 2019, the youth unemployment rate in Portugal was estimated at 18.3%, still above the European average, despite having had a significant reduction in Europe. The unemployment rate, in Portugal, in the fourth quarter of 2019 was 6.7%. However, in March 2020, the same source reveals that were created 2565 companies. In the perspective of Vell (2009), the increase in entrepreneurship levels leads to an increase in the progress of economic performance and the hiring of employees by new entrepreneurs; therefore, the increase in entrepreneurship levels leads to a decrease in unemployment. Consequently, there is an urgent need for entrepreneurship to develop a country’s social and economic market (Deli, 2011). Currently, it is recognized that women play an essential role in a country’s growth process and that their participation can strengthen economic acceleration (Micozzi and Lucarelli, 2016). Some authors (e.g., Silva et al., 2019) say that PsyResil can play an important role in motivating women who face adversity since the beginning of their entrepreneurial activities. In this sense, we expect that PsyResil can positively predict and mediate the effect on entrepreneurial intentions, and that the resilience will have a higher impact on women’s performance.

H7: For males and females—PsyResil has a significant positive effect on EI, which is not significantly different from each other.

H8: For males and females—PsyResil mediates the positive effect between PBC and EI, which effect is significantly stronger in females.

H9: For males and females—PsyResil mediates the positive effect between ATE and EI, and is not significantly different from each other.

For males and females: PBC (H10) and ATE (H11) positively increase PWB, one of the reasons that makes individuals more resilient to deal with the undertake process, having a positive effect in their EI.

**FIGURE 1:**
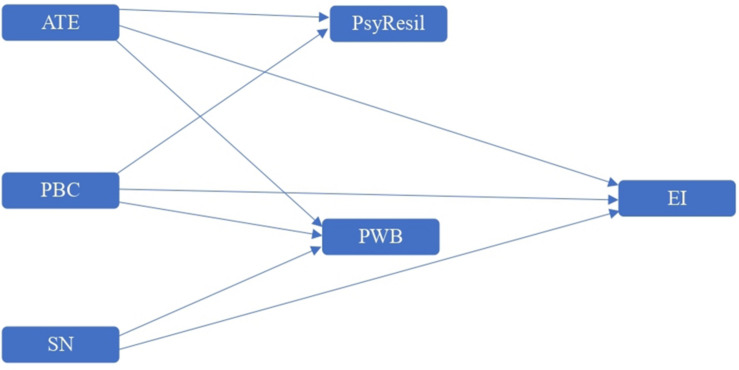
Structural model.

In view of the above, Figure 1 represents our structural model. Due to the influence of other variables present, we controlled its effect. Previous studies highlight the influence of previous work experience in relation to entrepreneurial intention (Arenius and Minniti, 2005), as well as the fact that parents have their own business (Kuckertz and Wagner, 2010).

## Materials and Methods

### Sample and Procedure

#### Sampling

The sample was collected by stratified sampling. It was proceeded as follows: (1) Despite being a small country, Portugal and the Islands have cultural and political variations and (2) the study did not specifically focus on business students, since different academic fields may also show differences in the entrepreneurial behavior. In order to increase the representativeness of the population, this study specifically aimed to reach as many possible geographic regions and academic fields.

The sampling was carried out between December 2018 and February 2019, through the collaboration of educational contacts from all Portuguese universities, which led the students to answer our questionnaire. Before the questionnaire, students should agree to an informed consent, where we specified the purpose of the study, as well as ensuring the protection of their data, which included anonymity and confidentiality.

All students received the questionnaire by e-mail, and they responded using an online platform. Participants were instructed on how to access the questionnaire and how to answer it. The questionnaire had no time limit to be answered, but the duration to fill it was estimated at 20 min approximately.

#### Participants

The study sample comprised of 644 university students from Portugal, representing the 18 districts and Azores Archipelagos and 21 universities and 7 polytechnic institutes. The age varied between 18 and the 64 years, with an average age of 25. Table 1 presents the demographic details of the sample by gender and academic field, and Table 2 presents the different regions of the country and islands. Women represent the majority (69%), while men represented 31%. Almost a third of the sample comes from courses in the Health field, namely, Medicine and Nursing (31.21%), followed by Management and Economy (18.32%) and Psychology (14.59%).

**TABLE 1:** Sociodemographic data.

**Variable**	**Female**		**Male**		**Total sample**	
	***N***	**%**	***N***	**%**	***N***	**%**
Gender	444	68.9	200	31.1	644	100
Previous work experience	203	45.72	107	53.5	310	48.14
Independent work experience	135	30.41	52	26.0	192	29.81
Independent work—mother	50	11.26	25	12.5	75	11.65
Independent work—father	66	14.86	50	25.0	116	18.01
Health	161	36.26	61	30.5	222	34.47
STEM	72	16.22	52	26.0	124	19.25
Law and humanities	39	8.56	47	24.0	86	13.36
Social sciences	75	16.89	19	9.5	94	14.59
Management and economy	79	17.79	39	19.5	118	18.32

*N = 644 students; universities = 29; age mean: 25.*

**TABLE 2:** Participants by regions.

**Region**	**Participants**
North	80
Center	193
Lisbon and Tejo Valley	174
Alentejo	83
Algarve	23
Azores Archipelago	91

#### Instruments

The Entrepreneurial Orientation Questionnaire (Sánchez-García, 2010) used in this study presents statements that must be answered in range metrics, that is, a Likert scale from 1 to 5. The scale has the specific objective of measuring entrepreneurial skills and related attitudes, and it is potentially useful for university students who must choose their professional future career. We used the following subscales, which will explain below, for which we also present the reliability of the original subscales: Perceived Behavioral Control (6 items, α = 0.884), Attitude toward Entrepreneurship (10 items, α = 0.834), Subjective Norm (4 items, α = 0.781), Psychological Resilience (9 items, α = 0.89), Psychological Well-Being (29 items, α = 0.90), and Entrepreneurial Intention (6 items, α = 0.936). This Questionnaire was administered to a sample of 1,810 university students from Spain, Portugal, Mexico, Brazil, and Argentina; for that reason, the original model of the scale was also validated for the Portuguese population.

Perceived Behavioral Control (PBC α = 0.942) is defined as the perception of the ease or difficulty of becoming an entrepreneur, the feeling of confidence and ability to control and carry out a behavior to create a company. There are some examples of the items: “Starting a business would be easy for me” or “I know how to develop an entrepreneurial process.” Both are respectively examples of self-efficacy and controllability.

Attitude Toward Entrepreneurship (ATE α = 0.964) is deeply connected to intentional and volitional behavior, beliefs, attitudes (Elfving, 2008), and a set of skills. ATE refers to the “degree to which a person has a favorable or unfavorable appraisal of the behavior under scrutiny” (Fini et al., 2012, p. 390). As an example, we highlight one of the items “I feel very competent and confident that I could identify market opportunities for a new business.”

Subjective Norm (SN α = 0.965) refers to the perceived social pressure to perform or not a behavior and the perception what the important people for the individual could think about the decision to become an entrepreneur. This variable is commonly measured by asking participants to what extent they think the relatives and close people that would support them in engaging in entrepreneurial activities (Ajzen, 2002; Liñán and Chen, 2009).

Psychological Resilience (PsyResil α = 0.952) can be considered as an ability to cope with adversities and recovering from adverse experiences, being a set of continuous behaviors, formed by the fusion of the following personal behavioral characteristics: flexibility, high motivation, perseverance, and optimism. This fact gives an entrepreneur with discernment the ability to adopt the application of different strategies to deal with a challenge until it is overcome (Margaça et al., 2020). We measure PsyResil by asking students, for example, “I think I can grow positively when facing difficult situations.”

Psychological well-being (PWB α = 0.961) is measured according to Ryff’s Psychological Well-being Scale reduced version (1995) (Ryff and Keyes, 1995). Well-being can be identified from the psychological resources that the individual has. There are cognitive, affective, and emotional processes that are globally described from six central dimensions to positive psychological functioning: acceptance of self, positive relationships with others, mastery environment, personal growth, life goals, and autonomy. There is the perception that well-being influences the cognitive processes involved in a conscious behavioral choices, such as decide to become an entrepreneur.

Entrepreneurial intention (EI α = 0.961) is a conscious state of mind that directs attention toward a specific goal or pathway in order to achieve the stated ambitions (Liñán and Fayolle, 2015). One of the items of assessment was “I will make any effort to start and develop my own business.”

In this study, three control variables were used: Spirituality, Previous Work Experience (PWE), and Independent Work Experience (IWE), the last two being dichotomous. Considering these last two variables, the literature points out that one of the factors that promote entrepreneurial intention is previous work experience (Carvalho and González, 2006). Some authors highlight Spirituality as being a strong predictor of a successful entrepreneur—in particular, someone who bases the company on the personal values. Spiritual intelligence focuses on skills that predict functioning, adaptation, and ability to produce valuable products and services. Accordingly, questions were asked such as: “When faced with an important decision, my spirituality plays absolutely no role (0) or it is always the primary consideration (10),” using the six-item Intrinsic Spirituality Scale by Hodge (2003)—which was translated and adapted for the Portuguese language (Spirit α = 0.981). This scale measures the degree to which spirituality functions as an individual’s master motive, for theistic and non-theistic populations, both within and outside of religious frameworks. The scale uses a sentence completion format to measure various attributes associated with spirituality; that is, an incomplete sentence fragment is provided, followed directly below by two phrases that are linked to a scale ranging from 0 to 10. The range provides with a continuum on which to reply, with 0 corresponding to absence or zero amount of the attribute, while 10 corresponds to the maximum amount of the attribute (Hodge, 2003).

### Statistical Procedure

In this study, to analyze the model and measure causal relationships, we used Structural Equation Modeling. For this, IBM SPSS Amos 23 and IBM SPSS 23 were used for the remaining analyses.

### Model Fit

In this study, the sample totalized 644 participants. According to Kline (2011), a typical sample size in studies where SEM is used is about 200 cases. Thus, the following indices were considered for model fit: the Comparative Fit Index (CFI), the Tucker–Lewis Index (TLI), the Adjusted Goodness of Fit (GFI), the Root Square Error of Approximation (RMSEA), and the Expected Cross Validation Index (ECVI). The adjustment index values are as follows: CFI > 0.90; GFI > 0.95; and RSMEA < 0.05 (Hair et al., 2010); TLI > 0.90 (Awang, 2012); although the ECVI does not have specific threshold indexes, it is assumed that the lower the index, the better the fit and the better the model can predict the future covariance of the sample (Browne and Cudeck, 1992). In order to demonstrate how much of the variation of the independent variables is explained by the predictors, multiple squared correlations (R2) were performed.

### Direct, Indirect, and Moderation Effects

The Maximum Likelihood Estimate was performed to calculate the coefficient and significance of the direct effects. In order to estimate the mediation effects and group differences, Bootstrap was used with 2000 interactions and 0.95 bias correction. The product or the difference between the unstandardized regression weights was considered, on the mediation or moderation path, to test whether the effect between the variables is statistically significant, at a 95% confidence level. The alpha was *p* < 0.05 for statistical significance.

### Mean Comparison Between Genders

The *t*-test statistic was used to calculate and compare the mean difference between genders. In order to observe the homogeneity of the variables (>0.05), we used the Levene test.

## Results

### Model Fit

The model adjustment indexes for SEM obtained in the study were CFI = 0.912; TLI = 0.901; GFI = 0.976; RSME = 0.04; and ECVI = 0.445. According to the index of adjustment values described above (Browne and Cudeck, 1992; Hair et al., 2010; Awang, 2012), our model presents a good fit and above the common standards, which means that the proposed model accounts for the correlations between the variables proposed in the data set. R2 values are also adequate, explaining in females 77% and in males 75% of the variance of the dependent variable. Pearson correlations can be found in Table 3 and highlight a strong and significant correlation of ATE with entrepreneurial intention, which corresponds what the literature point out. The results achieved allow us to underline the required theoretical coherence, thus, we proceed to test the remaining hypotheses.

**TABLE 3:** Pearson correlation analyses.

	**1**	**2**	**3**	**4**	**5**	**6**	**7**	**8**	**9**
1. PBC	1								
2. ATE	0.880**	1							
2. SN	0.908**	0.842**	1						
4. PsyResil	0.831**	0.854**	0.840**	1					
5. PWB	–0.006	–0.005	–0.006	–0.005	1				
6. Spirit	0.849**	0.869**	0.961**	0.916**	0.014	1			
7. PWE	–0.016	–0.035	–0.016	–0.031	–0.040	–0.026	1		
8. IWE	0.039	0.037	0.034	0.039	–0.064	0.042	–0.045	1	
9. EI	0.814**	0.849**	0.823**	0.916**	–0.002	0.921**	–0.019	0.033	1

**Correlation is significant at the 0.01 level (2-tailed).

### Regression Weights

It is important to highlight the weight of each regression to understand how each variable interacts individually, before elaborating the path model. Thus, we demonstrate these values for both genders in Table 4.

**TABLE 4:** Regression weights by gender.

	**Females**			**Males**		
	**B**	**SE**	**ρ**	**B**	**SE**	**ρ**
PWB←PWE	0.378	0.409	0.299	0.152	0.261	0.438
PWB←IWE	0.122	0.048	0.389	0.039	0.126	0.585
PWB←ATE	0.173	0.035	***	0.243	0.068	0.004**
PWB←PBC	0.086	0.028	0.068	0.198	0.055	0.003**
PWB←SN	0.124	0.036	0.022*	–0.022	0.058	0.781
PsyResil←PBC	0.188	0.041	***	0.021	0.056	0.789
PsyResil←ATE	0.150	0.055	***	0.222	0.097	0.171
PsyResil←SN	0.132	0.024	0.023*	0.326	0.078	0.231
PsyResil←Spirit	0.161	0.055	***	0.372	0.078	***
PsyResil←PWB	0.199	0.038	***	0.176	0.069	0.031*
PsyResil←IWE	–0.221	0.511	0.399	0.041	0.311	0.896
EI←PBC	0.623	0.039	***	0.734	0.069	***
EI←ATE	0.146	0.044	0.003**	0.439	0.089	***
EI←SN	0.335	0.048	***	0.186	0.085	0.031*
EI←PWE	–0.042	0.059	0.546	–0.133	0.213	0.212
EI←IWE	–0.179	0.499	0.697	0.062	0.481	0.787
EI←PsyResil	0.123	0.042	0.039*	0.064	0.096	0.630
EI←PWB	0.301	0.049	***	0.150	0.078	0.123
EI←Spirit	0.408	0.042	***	0.398	0.051	***

Maximum likelihood estimation; B: unstandardized estimates; ***p = 0.001 or less; is significant at the <0.05 value, **p < 0.01; *p < 0.05.

The three exogenous variables of our model significantly predict the students’ entrepreneurial intention, with the PBC presenting a stronger regression value, for both female and male. There is a significant difference between coefficients from the other exogenous variables on gender. For females: *p* = 0.001 when to compared to ATE, and when compared to SN *p* = 0.001; and for males: when compared to ATE *p* = 0.017 and *p* = 0.001 when compared to SN. No statistically significant differences were found between groups (for PBC *p* = 0.389, for SN *p* = 0.145, and for SN *p* = 0.376). However, females reach higher coefficients on SN, and males on PBC and ATE. That is, the perception of these variables of both genders affects their intentions, although not significantly different from each other.

For male, PBC and ATE have a statistically significant regression to PWB. However, none of them affect significantly the male students’ PsyResil. In the case of female, the PBC has not a significant regression in the PWB, and in the case of PsyResil both (PBC and ATE) has a significant regression. SN has no significant impact in male for PWB and PsyResil, but the opposite is true for females.

PsyResil impacts significantly entrepreneurial intentions in females, but not in males. Similarly, the PWB effect is drastically stronger and significant on entrepreneurial intentions in females, but not in males.

Regarding the other control variables, Spirit effect is drastically stronger and significant on entrepreneurial intentions and PsyResil in both females and males. Interactions with PWE and IWE presented non-significant (e.g., PWB) and/or negative effects. Both PWE and IWE impact negatively in females’ intentions; in the case of males, only PWE has a negative effect.

### Path Model Effects

When females have a favorable and elevated perception to achieve an entrepreneurial behavior, this increases their well-being and the entrepreneurial intention. That is, the PWB mediates a very positive and significant effect between ATE and EI. The PWB also mediates positively and significantly the relationship between SN and EI.

PsyResil mediates a significant effect between PBC and Intention in females, but not in males. Also mediates a significant effect between ATE and Intention in females, but not in males. We ran a serial mediation path and found that ATE positively affects (1) PWB, which affects (2) PsyResil and that in turn affects (3) EI, just in females. These results highlighted that ATE is a determining factor for females to achieve their entrepreneurial activities. Table 5 demonstrates the results obtained from path model by gender.

**TABLE 5:** Effects for path model by gender.

	**Females**				**Males**			
	**Effects**		**Confidence interval**		**Effects**		**Confidence interval**	
	**β**	**ρ**	**LB**	**UB**	**β**	**ρ**	**LB**	**UB**
PBC → EI	0.522	***	.	.	0.623	***	.	.
ATE → EI	0.122	0.003**	.	.	0.244	0.007**	.	.
SN → EI	0.211	***	.	.	0.176	0.041*	.	.
PBC → PsyResil → EI	0.107	0.004**	0.010	0.058	0.002	0.634	–0.011	0.032
ATE → PsyResil → EI	0.014	0.024*	0.002	0.034	0.008	0.421	–0.011	0.072
PBC → PWB → EI	0.014	0.066	–0.001	0.037	0.032	0.048*	0.000	0.092
ATE → PWB → EI	0.044	***	0.017	0.074	0.044	0.066	–0.001	0.098
SN → PWB → EI	0.018	0.015*	0.004	0.040	–0.003	0.834	–0.042	0.032
PBC → PWB → PsyResil	0.013	0.052	0.000	0.033	0.043	0.022*	0.006	0.097
ATE → PWB → PsyResil	0.044	***	0.012	0.058	0.042	0.038*	0.004	0.110
PWB → PsyResil → EI	0.022	0.023*	0.004	0.047	0.011	0.367	–0.023	0.064
PBC → PWB → PsyResil → EI	0.004	0.060	0.000	0.005	0.004	0.265	–0.004	0.015
ATE→ PWB→ PsyResil→ EI	0.006	0.017*	0.001	0.011	0.004	0.322	–0.005	0.018

β: standardized estimates; ***p = 0.001 or less; *p* is significant at the <0.05 value. **p < 0.01; *p < 0.05. Indirect effects: Bootstrapping: 2000 iterations and 0.95 bias-corrected. Direct effects: maximum likelihood estimation. LB is the lower bound of the confidence interval; UB is the upper bound of the confidence interval.

### Mean Comparison Between Genders

Table 6 indicates the average of each variable by gender, and the results are obtained in *t*-test analysis. The biggest responses’ difference we found concerns EI, with a mean difference of –0.234 (significant, *p* = 0.004), and the smallest difference concerns ATE, with a value of mean difference of –0.045 (not significant, *p* = 0.543). Considering that they are university students, regardless of gender, we conclude that the fact that there is no difference between them is due to the ease of access to resources for promotion and improvement of their skills and competences, which could be useful in the case that they become entrepreneurs. Cohen’s d is an appropriate effect size for the comparison between two means. When we calculated this test for differences between means, was found that only the PWB and EI reached acceptable effect size values, *d* = 0.48 (medium effect size) and *d* = 0.241 (small effect size), respectively (Cohen, 1988).

**TABLE 6:** Variables’ mean by gender.

**Variable**	**Mean by gender**				***t*-test**		
	**Gender**	**Mean**	**SD**	**SE**	***t***	**ρ**	**Mean dif.**^a^
PBC	Male	3.585	0.872	0.062	–2.342	0.019	–0.160
	Female	3.745	0.769	0.037			
ATE	Male	3.563	0.965	0.068	–0.609	0.543	–0.045
	Female	3.608	0.820	0.039			
SN	Male	3.664	0.975	0.068	–2.127	0.034	–0.166
	Female	3.830	0.889	0.042			
PWB	Male	3.632	0.367	0.026	5.685	0.001	0.178
	Female	3.454	0.368	0.017			
PsyResil	Male	3.538	0.878	0.062	–0.784	0.433	–0.055
	Female	3.593	0.798	0.038			
EI	Male	3.542	1.006	0.071	–2.866	0.004	–0.234
	Female	3.776	0.937	0.044			

*t-test statistic for equality of means by gender. For *t*-test = equality of variance is assumed in all variables: Levene’s test = *p* > 0.05; 95% confidence interval for lower and upper values.*

*^*a*^Mean difference positive value means males score higher, negative value means females score higher.*

Contrary to what would be expected and according to several sources, our sample contradicts the common tendency for males to score higher and significantly in EI, with females presenting higher and more significant values. Males only score higher and significantly for the PWB. In other words, their psychological sustainability allows the creation, intrinsically and more effectively than women, of the necessary social milieu to take the step toward entrepreneurship.

## Discussion

### Discussion of the Results

Several studies (e.g., Bohnenberger et al., 2007) suggest that entrepreneurial behavior and the development of entrepreneurship are influenced, above all, by the individuals’ first social group—the family. However, entrepreneurial behavior can also be learned (Colette, 2013) and, therefore, the influence of entities/organizations is evident, as is the case with Universities, which support young people, transmitting knowledge and skills. In addition to the academic context, in Portugal, there are business initiatives, in the form of associations or organizations (e.g., ANJE—National Association of Young Entrepreneurs), which play a crucial role in promoting young people’s attitude toward entrepreneurship. In this study, it was possible to verify that there are no statistically significant differences in the attitude toward entrepreneurship between both genders’ students, and the attitude positively impacts the intention to undertake.

Entrepreneurship is increasingly becoming an alternative means of entering the labor market; the result of European and Portuguese directives aimed at universities (European Commission, 2013; Marques, 2015). On the one hand, there is consensus that the University can be seen as a means of promoting entrepreneurship among students, and for another the study area is not relevant. For instance, in the study by Sieger et al. (2014) it is evident that the students of management and economics have the highest levels of entrepreneurial intention; a study by Teixeira (2008) pointed out that Portuguese Pharmacy students were more entrepreneurial, yet another study with Portuguese students reveals that Social Science students score at the same level as Management Science students, regarding their desire to become entrepreneurs (Santos et al., 2010). As discussed in previous studies, it is clear that entrepreneurial skills are not a unique characteristic to the business students (e.g., Ward et al., 2019; and many other). However, exposure to knowledge and learning useful skills for creating an own business have also revealed slight impacts on intention. Thus, the university educational environment becomes an equal means in terms of gender, as it eliminates nuances of discrimination between them. A reflection of this is the fact that the sample does not reveal significant differences. Rauch and Hulsink (2015) suggest that entrepreneurship education is effective in entrepreneurial intention, and although there are no differences between both genders, this aspect allows students to increase their attitudes and perceived behavioral control.

Females’ perceived social pressure to perform or not a behavior is higher; however, this variable predicts entrepreneurial intentions, which is not significantly different between both genders. This finding is corroborated by Robledo et al. (2015), who point out as a possible explanation the fact that these results can be related to the larger influence reference groups have on women in comparison with men. These authors also acknowledge that this result could be indicating that stereotypes related to the male gender domain for a greater intention to be self-employed may be disappearing, except for the greater influence of the social pressure perceived by women and their higher affiliation needs, which means they are more likely to conform to majority opinions (Morris et al., 2005; Robledo et al., 2015).

Psychological well-being mediates this effect on intentions in females, but not in males. This may suggest that positive relationships with others, personal mastery, autonomy, a feeling of purpose and meaning in life, and personal growth and development could be relevant under determining intentions in women and it may be a potential explanation for the influence (or lack thereof) in the intentions of both genders.

Social recognition is very important for female students (Ferri et al., 2018), which means that the opinions of parents, relatives, friends, and important others might be influential in the decision-making process of becoming an entrepreneur (Minniti, 2009; Ferri et al., 2018). Today, there is a consensus that the participation of women in entrepreneurship is a major factor in development (Lepeley, 2019); therefore, there is a critical element to promote inclusive development (Gallup International Labor Organization, 2017) and to increase happiness (Helliwell et al., 2019) and diversity (Hunt et al., 2018), with the active participation of women, particularly based on implicit impact of the multiplier effect. The well-being of women who want to become entrepreneurs correlates highly with attainment of work–life balance, and work engagement reaching to high levels of productivity that unleash the multiplier effect of their actions, propelling sustainable business ventures in developed and developing nations (Lepeley, 2019). These results may reflect the creation of programs to support entrepreneurship exclusively for women, in Portugal. For example, the FAME program (Advanced Training for Women Entrepreneurs), which started in 2001, is based on three crucial pillars: training, consultancy, and financial support (Instituto para o Fomento e Desenvolvimento do Empreendedorismo em P ortugal [IFDEP], 2020).

The findings revealed that perceived behavioral control predicts significantly stronger than subjective norms on entrepreneurial intentions in both genders, and it did not have an effect significantly different between males and females. Perceived behavioral control is considered as the most controversial construct in the Theory of Planned Behavior for two reasons: the inconsistency in the empirical findings related to its influence on intention and in the disagreement regarding its conceptualization and operationalization (Yap et al., 2013), and it is often associated with self-efficacy (Zhao et al., 2010). Although the difference in the mean between both genders is statistically significant, the values are not high. However, the importance of perceived control is desirable, as it corresponds to a self-assessment of your knowledge and skills regarding the creation of an entrepreneurial activity (Fragoso et al., 2020). Hence, as acknowledge by several authors, the existence of educational programs linked to entrepreneurship increases self-efficacy and reinforces the intention to become an entrepreneur (Fragoso et al., 2020).

The results of female students demonstrated that the more positive the salient beliefs regarding behavior and the personal assessment of its consequences, the higher their ability to resist an adverse situation, which would also affect their entrepreneurial intention. The same occurred with psychological well-being. This may mean that reaching a state of equilibrium affected by challenging and rewarding life events allows them to define more clearly a path toward entrepreneurship. This is a remarkable finding—most studies present a much stronger relationship between attitude and intention toward males (Díaz-García and Jiménez-Moreno, 2010; Paço et al., 2015; and many others). In line with our results is the study by Trzcinski and Holst (2012), who stated that well-being was stronger in male than in female, regarding their work. Thus, males’ perceptions of their ability to perform a given behavior allied to a state of balance makes men more ability to start an entrepreneurial activity.

Resilience is cited as an essential and decisive factor for the entrepreneurs’ success and their company (e.g., Hedner et al., 2011). However, there are few studies that have included this construct in the analysis of the entrepreneurial process (Fisher et al., 2016). These results indicate that psychological resilience mediates the relationship between PBC and entrepreneurial intention, and ATE and entrepreneurial intention in women. The perceived ability to face challenges and overcome obstacles, resulting from an entrepreneurial process, allows them to be more persevering. That is, the dynamics of resilience can assist this process, facilitating an adequate interpretation of adversities and the development of coping skills (Jing et al., 2016; González-López et al., 2019; Santoro et al., 2020). Positive attitudes toward risk due to certain behavior as part of the successful entrepreneurial activity process are associated with the resilience of entrepreneurs, complementing other formal professional capabilities (González-López et al., 2019). A study conducted in Portugal with a sample of university students reveals that they know and adopt coping strategies in the face of stressful events, namely, those concerning “Acceptance of Responsibility” and “Planned Resolution of the Problem” (Silva et al., 2020). It is also important to note that individuals’ perceptions with regard to the presence or the absence of the necessary resources and opportunities to develop the conduct that influences their ability to overcome any obstacle that may arise. The Portuguese economic crisis broke out in 2008 and persisted until 2013, which triggered a period of rising unemployment. Several studies prove that entrepreneurship is fundamental for socioeconomic development, taking the economy forward. Although unemployment seems to be a negative factor, in Portugal there is a program that allows the beneficiaries of the unemployment benefit to receive the total amount to which they are entitled to start their own business (Instituto do Emprego e Formação Profissional, 2019). For example, between January 2010 and July 2019, projects for 21630 beneficiaries were approved, creating more than 20500 new businesses and consequent jobs (Instituto do Emprego e Formação Profissional, 2019).

### Theoretical Contributions

Generally, our study contributes to the literature on entrepreneurship, in particular because it creates causal relationships between two psychological variables and the entrepreneurial intention of Portuguese female and male students. Contrary to the most studies concluded, our findings revealed a greater propensity for women to initiate an entrepreneurial activity. According to the conclusions of the Mastercard Index of Women Entrepreneurs (2019), held in 58 countries in five regions of the globe, Portugal is the 10th country in the world with the best opportunities and support conditions for women to prosper as entrepreneurs. This report reveals that Portugal has a high rate of women business owners (30.2%), higher than Spain (29.9%), for example. In general, this high representation of women entrepreneurs appears to be correlated positively with high business leadership, higher education, and entrepreneurial supporting factors. Women face pressure better and are more resilient, able to adapt to new challenges, and more flexible than men.

This study also contributes to the understanding of how soft skills, such as resilience, influence the decision-making process to start an entrepreneurial activity and how it varies between the both genders. The inclusion of resilience in an entrepreneurial intention model provides a deeper understanding of this process and the variations between males and females and highlights possible factors to consider in the development of more comprehensive models (Krueger et al., 2000; Fayolle and Liñán, 2014). This study highlighted two issues: it is not the category of business students who have the greatest entrepreneurial intention, and it is not males who have the highest levels of intention. Few studies have been done on the relationship between resilience and entrepreneurial intention in relation to university students. Thus, we believe that, on the one hand, studies on intention should cover all areas of study and, on the other, the introduction of resilience training (González-López et al., 2019) and the importance of psychological well-being to education programs for entrepreneurship.

### Practical Implications

The study reveals that Portuguese university students face entrepreneurship as a possible path to the job market, particularly in the female students. The finding reinforces also the importance of the role of higher education institutions, and other public or private institutions, in improving entrepreneurship. In this way, these findings can be useful for policymakers and institutions responsible for creating entrepreneurship training programs, as well as its inclusion in the curricular structures of the various learning cycles—from secondary education to higher education—in order to influence both the antecedents of the planned behavior model and entrepreneurial skills. The design thinking method reinforces self-confidence, allowing the individual to learn to deal with subjective threats, which also improves the development of self-efficacy (González-López et al., 2019). The importance of emotional aspects can be understood through personal testimonies and seminars aimed at planning entrepreneurial careers.

Also, an important measure is to monitor these programs, in a longitudinal way, in order to guarantee a real evaluation of the results. It is essential to strengthen the viability of the future entrepreneur ideas together with stakeholders and sponsors, in order to expose students to the idea that entrepreneurship is a viable path for self-employment. Fortunately, in Portugal, the process of creating a company is gradually less bureaucratic, which shows that entrepreneurship is being supported by various entities in the country, namely, with programs aimed at women and young people.

The female presence in management has also grown in most sectors. Moreover, it is in small businesses that the percentage of management positions held by women is highest, with 30.9%—in many cases the result of their own entrepreneurial initiative. Hence, the institutions have the responsibility to combat the misinformation that exists in the female population regarding entrepreneurship and the creation of a business, ending the female stigma in the business world.

The presence of initiatives like Web Summit, in Portugal since 2016, brought a number of obvious benefits, such as conferences with world leaders, the presence of investors, the public exposure of innovative technologies, and the consolidation of many national entrepreneurs and startups. This set of factors contributes to the dynamism, training, and visibility of the Portuguese entrepreneurial ecosystem. The fact that Portugal welcomes this type of initiative allows to explore the virtual side of the spirit of entrepreneurship and innovation in the country.

Universities are seen as points of reference in the reconstruction of the conception of science, as well as promoters of innovation in the economic development of nations. The triple-helix thesis (Etzkowitz and Leydesdorff, 2000) analyzes the relations between the University, Industry, and the State. This theory highlights that the University can (and should) play an increasingly important role in innovation in the context of knowledge-based societies. Thus, the concept of academic entrepreneurship rose, resulting from research carried out at universities, and it presents itself as their third mission. In Portugal, incentives have been created to encourage the use of Intellectual Property rights, to ensure legal protection for products and/or technologies, namely, the creation of 22 offices, 10 of them being based in universities. For example, the University of Porto created a Portal with the objective to support the innovation value chain, promoting the transfer of knowledge and strengthening the University’s connection to companies, also through the incubation or financing of startups or business ideas. Since 2007, it has supported more than 550 business projects, welcomed 186 business ideas, and registered 73 graduated companies, that is, startups that were born in its facilities, developed, and made its leap into the world. We consider that the perception of supporting programs inside and outside the University leads students to believe that entrepreneurship is a possible path in the professional career option, without disregarding their idiosyncrasies.

### Limitations and Suggestions for Future Research

The current study presents certain limitations that could be addressed in future studies in this field. Our study used variables that allow us to evaluate, in part, the perception of male and female students regarding entrepreneurship in the Portuguese context. Despite knowing that the research brought promising results in this field of study, we conclude that it is important to introduce other variables and theories that indicate a more reliable entrepreneurial profile. Thus, it is necessary to study a more complete model that can extend the evaluation of characteristics such as creativity and innovation, in parallel with, for example, the Theory of Basic Needs and the Theory of Self-Determination. Studying this, it is possible to understand why people are naturally curious and intrinsically motivated to perform an activity, and not through extrinsic motivators, such as remuneration.

Regarding the sample, we identified one that is not gender-equitable, which can skew the results. In future studies, it is important to consider a sample where the both genders are represented equally. Another issue is related to the university context, considering that we only evaluated Portuguese students. In order to better assess and contrast the intention of the university population, it is important to include other countries to understand whether cultural and context differences influence entrepreneurial intent or not. The study considered a sample of students from different academic years. Given that the characteristics and skills of entrepreneurs tend to fluctuate over time, a longitudinal survey could be carried out in order to consider whether the continued exposure to knowledge and to programs effectively leads students to create their own business.

Vamvaka et al. (2020) found that the construct of perceived behavioral control is better described by a two-factor solution, with the one representing perceived controllability and the other perceived self-efficacy. Thus, in future studies it is important to evaluate these two variables, in order to obtain more reliable results.

## Data Availability Statement

The original contributions presented in the study are included in the article/supplementary material, further inquiries can be directed to the corresponding author/s.

## Author Contributions

CM contributed to writing, sampling, statistics, and discussion. BH-S contributed to writing and discussion. JS-G carried out the statistics and discussion. GC discussed the article. All authors contributed equally to the preparation of this article and approved the final version submitted.

## Conflict of Interest

The authors declare that the research was conducted in the absence of any commercial or financial relationships that could be construed as a potential conflict of interest.
